# Determinants of attendant satisfaction with oncology service quality in Tamil Nadu, India: a cross-sectional study

**DOI:** 10.1038/s41598-026-58350-z

**Published:** 2026-06-16

**Authors:** J. Benita Evangalyne, T. Paul Robert, K. S. Rajkumar, J. Anju

**Affiliations:** 1 Department of Industrial Engineering, College of Engineering, Anna University, Chennai, Tamil Nadu India; 2 Department of Surgical Oncology, PSG Hospitals, Coimbatore, Tamil Nadu India; 3 PSG Hospitals, Coimbatore, Tamil Nadu India

**Keywords:** Cancer care, Attendant satisfaction, Service quality dimensions, Demographic differences, Structural equation modelling, Cancer, Health care, Oncology

## Abstract

Attendants are continuously involved in coordinating and supporting cancer care, yet their service quality perceptions are rarely modelled as determinants of satisfaction. This study investigates the association between demographic factors and service quality dimensions related to attendants’ satisfaction in oncology hospitals. A cross-sectional survey using a structured questionnaire was conducted among 480 attendants (accompanying patients) across oncology hospitals in Tamil Nadu, India. Service quality was assessed using six dimensions: tangibility, responsiveness, empathy, assurance, service reliability, and process reliability. Differences across demographic groups were evaluated using non-parametric statistical tests. Partial Least Squares Structural Equation Modeling (PLS-SEM) was applied to examine the relationships between service quality dimensions and attendant satisfaction, followed by Importance-Performance Map Analysis (IPMA) to prioritize improvement areas. Significant differences in service quality perceptions were observed across gender, age, education, residential status, financing category, and hospital visit frequency, while income and occupation did not show meaningful effects. Tangibility (β = 0.374), responsiveness (β = 0.313), and empathy (β = 0.196) demonstrated significant positive associations with satisfaction, collectively explaining 80.9% of its variance (R^2^ = 0.809), whereas assurance, service reliability, and process reliability were not significantly associated with satisfaction. IPMA result indicate that empathy represents a key area for refinement, while tangibility and responsiveness demonstrate high importance alongside strong performance, suggesting areas to be maintained. Overall, the findings provide insights into factors associated with attendant satisfaction, improving patient-centered oncology services.

## Introduction

Globally, cancer incidence remains high, with 19.3 million new cases recorded in 2020 and projections indicating an increase to 27.5 million by 2040^1^. Despite significant medical and technological advancements, cancer remains the second leading cause of death globally, with low- and middle-income countries accounting for nearly 70% of related mortality. In India, this burden is particularly alarming: cancer was the fourth leading cause of death in 2017, and the country recorded 1.15 million new cases in 2018, with incidence expected to double by 2040 due to demographic shifts^2^. Although India reports fewer new cancer cases than many high-income countries, its mortality burden is disproportionately high, with cancer related deaths exceeding those in the United States by nearly 30%^3^. This elevated mortality is further compounded by the concentration of cancer care infrastructure in urban tertiary hospitals, resulting in challenges related to accessibility, timeliness, and equitable provision of high-quality care^4^. Ensuring quality in oncology services is therefore a critical public health priority^5^. Beyond its clinical implications, cancer care is inherently complex and emotionally intensive, requiring sustained interaction between patients, families, and healthcare systems. In such high-contact and high-emotion services, ensuring superior service quality is critical, as it becomes a critical determinant of satisfaction, treatment adherence, and overall healthcare outcomes.

Service quality has been widely recognized as a key determinant of satisfaction in healthcare. Although service quality and satisfaction in healthcare have been widely examined, existing research in oncology care remains predominantly patient-centric. Studies conducted across different countries have extensively evaluated oncology service quality from the patient perspective. Research conducted in the United States by Gupta et al. demonstrated that patient satisfaction with service quality significantly influences treatment outcomes^6^, while studies from Sweden by Kittang et al. highlighted the importance of professional care, safety, and individualized attention in shaping patient perceptions^7^. Similarly, Zarei et al. in Iran^8^, and Mahran and Al Nagshabandi in Saudi Arabia^9^ have examined patient satisfaction as a key indicator of oncology service quality. Recent evidence from low- and middle-income countries further emphasizes the role of communication and care coordination in determining patient experience^10^.

While these studies provide valuable insights, they predominantly adopt a patient-centric perspective, thereby overlooking other key stakeholders involved in cancer care. In practice, oncology care extends beyond the patient to include attendants such as family members or caregivers who accompany patients throughout diagnosis, treatment, and follow-up^11^. Attendants play a vital role in coordinating appointments, managing hospital procedures, facilitating communication with healthcare providers, and offering emotional and logistical support^12^. Due to their continuous engagement with healthcare systems, they develop a comprehensive understanding of service delivery processes and are well positioned to evaluate the services provided.

Despite this substantial body of patient-focused research, the perspectives of attendants who accompany patients remain largely underexplored in empirical research. A recent scoping review by Nila et al. reports that nearly 79% of healthcare experience studies focus exclusively on patients, with minimal inclusion of caregivers and limited approaches to assess their perspectives^13^. Existing caregiver studies, primarily conducted in developed countries, have largely focused on caregiver burden and psychological outcomes, and do not clearly define caregivers as accompanying attendants or examine their role in evaluating healthcare service quality.

This gap is particularly pronounced in the Indian context, where family members are deeply involved in caregiving and interact extensively with healthcare systems. Despite their significant role, study specifically examining attendant satisfaction with oncology service quality in India remains underexplored, and no empirical evidence exists on how their perceptions are shaped or identifying the service dimensions that most strongly influence their satisfaction. Furthermore, demographic characteristics are known to shape healthcare perceptions, yet their influence on attendant evaluations remains insufficiently explored.

Therefore, a clear research gap exists in examining attendant satisfaction with oncology service quality, particularly within the Indian healthcare context, current assessments of oncology service quality provide only a partial understanding of healthcare experiences, as they largely exclude attendant perspectives and overlook demographic variability. Addressing this gap is essential to develop a more comprehensive and inclusive evaluation of service quality in cancer care.

To bridge this gap, the present study focuses on attendants accompanying cancer patients in India and examines their perceptions of oncology service quality. Specifically, it investigates the influence of demographic characteristics on attendant perceptions and identifies the key service quality dimensions that determine their satisfaction. Accordingly, the study is guided by the following research questions:

- RQ1 How do demographic characteristics influence attendant perceptions of oncology service quality?
- RQ2 What are the key service quality dimensions influencing attendant satisfaction in oncology care?

This study contributes by extending the evaluation of oncology service quality beyond the patient perspective and incorporating attendants as active stakeholders. It offers a more holistic understanding of healthcare experiences and provides actionable insights for improving attendant-inclusive cancer care.

## Research hypotheses development

**Figure 1:**
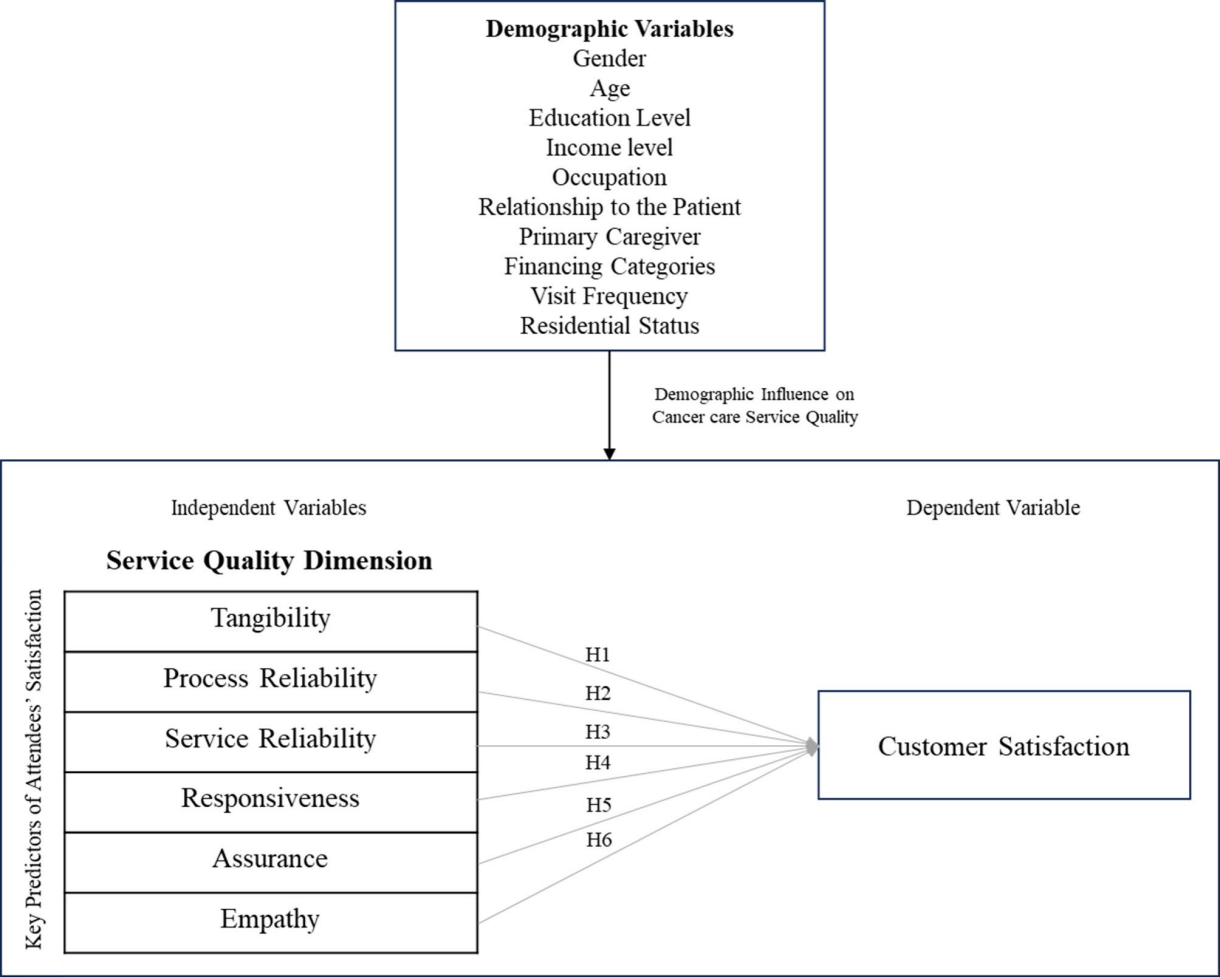
Conceptual framework of cancer care service quality with hypotheses development and attendant demographics.

Figure 1 depicts the conceptual model that connects the study’s hypotheses.

### Hypothesis 1

Demographic Differences in Service Quality Perceptions.

Understanding whether service quality perceptions differ across demographic groups is essential for ensuring equitable healthcare delivery. Studies report variations in perceived service quality across different demographic categories. Braimah^14^, examining older adults with low income in Ghana, identified that socio-economic and demographic factors contribute to variations in healthcare utilisation dissatisfaction across demographic groups. Mini et al.^15^, in a study among inpatients in a public tertiary care hospital in Kerala, India, reported that socio-demographic factors such as gender, marital status, and travel time are significantly associated with patient satisfaction. In addition, Devaraju^16^, in a study among patients in primary health centres in Telangana, India, found significant differences in satisfaction across age, gender, education, and income groups. Likewise, Batta et al.^17^, in a study among patients in a tertiary care hospital in North India, confirmed that demographic variables such as age, gender, and education are significantly associated with variations in patient satisfaction and perceived service quality. These findings collectively indicate that service quality perceptions are not homogeneous but vary across demographic categories.

Based on this evidence, the present study proposes the following hypothesis to examine whether attendants’ perceptions of cancer care service quality differ across demographic categories. Hypothesis 1 as follows,

### (H_0_)

null hypothesis stated that there is no significant difference in cancer care service quality perceptions across demographic groups.

### (H_1_)

alternative hypothesis stated that there are significant differences in cancer care service quality perceptions across demographic groups.

### Hypothesis 2

Impact of Service Quality on Customer Satisfaction.

In healthcare service research, service quality and customer satisfaction are considered fundamental constructs, widely used to evaluate service performance and user experiences. In the healthcare sector, positive relationships have consistently been observed between service quality dimensions and overall customer satisfaction^18–22^. Studies demonstrate that key service quality dimensions such as reliability, responsiveness, assurance, empathy, and tangibility significantly influence patient satisfaction across different healthcare settings which collectively capture the functional and relational aspects of service delivery. Service quality reflects users’ perceptions of how well a service meets or exceeds their expectations, while customer satisfaction represents their overall evaluation of the service experience. These constructs are particularly critical in healthcare due to the intangible and interaction-intensive nature of services, where both technical outcomes and interpersonal interactions shape user perceptions.

Empirical studies further reinforce this relationship by identifying service quality as a key antecedent of satisfaction. Alfatafta et al. (2025, Jordan)^23^ reported that service quality dimensions significantly influence patient experience and satisfaction, with responsiveness, assurance, and empathy playing a crucial role. Similarly, Yunus et al. (2024, Malaysia)^24^ found a strong positive impact of healthcare service quality on patient satisfaction, emphasizing the importance of reliable and efficient service delivery. This association is further evident in oncology care settings. Al-Awadi et al. (2024, Kuwait)^25^ demonstrated that patient satisfaction is strongly shaped by service quality factors such as communication, coordination, and provider interaction. Likewise, Vastardi et al. (2024, Italy)^26^ showed that improvements in service quality in oncology day care significantly enhance patient satisfaction and overall quality of life.

As healthcare services are delivered through continuous interactions, satisfaction is largely formed through service encounters, making the role of service quality even more critical. This is particularly relevant in oncology care, where prolonged and emotionally intensive interactions lead to higher expectations and sensitivity to service performance. In such contexts, service quality dimensions play a vital role in shaping attendant experiences and satisfaction^27^.

Based on this theoretical and empirical foundation, service quality is conceptualized as a key antecedent of satisfaction in the present study. Similarly, this present study deals with the expected relationship between service quality attributes on attendant satisfaction for cancer care services.

### Hypothesis 2

Each of the service quality dimensions has a positive and significant influence on the satisfaction experienced by attendants.

The auxiliary hypotheses corresponding to the main hypothesis are presented below:

### H1

Tangibility, as a dimension of perceived cancer care service quality, has a positive and significant effect on customer satisfaction.

### H2

Process reliability, as a dimension of perceived cancer care service quality, has a positive and significant effect on customer satisfaction.

### H3

Service reliability, as a dimension of perceived cancer care service quality, has a positive and significant effect on customer satisfaction.

### H4

Responsiveness, as a dimension of perceived cancer care service quality, has a positive and significant effect on customer satisfaction.

### H5

Assurance, as a dimension of perceived cancer care service quality, has a positive and significant effect on customer satisfaction.

### H6

Empathy, as a dimension of perceived cancer care service quality, has a positive and significant effect on customer satisfaction.

This study will test the following hypotheses, which correspond with each of the research questions.

### Conceptual framework

Accordingly, this study proposes a conceptual framework, as depicted in Fig. 1. This framework incorporates demographic characteristics as influencing factors and conceptualizes oncology service quality from the attendants’ perspective across six key dimensions. The demographic categories considered in the study are presented in Fig. 1. The service quality dimension includes: (1) Tangibility, referring to physical aspects like facilities and equipment; (2) Process Reliability, emphasizing the consistency of service procedures; (3) Service Reliability, reflecting the dependability of care; (4) Responsiveness, concerning timeliness and attention to patient needs; (5) Assurance, representing patient confidence in provider expertise and courtesy; and (6) Empathy, denoting understanding and compassion during service delivery.

## Study design and methods

### Study setting

To test the proposed hypotheses, this study adopted a cross-sectional research design conducted across oncology centers in Tamil Nadu, India, from September 2024 to October 2025. Data were collected from 480 attendants accompanying cancer patients using a structured questionnaire administered through face-to-face interactions in cancer care units to assess their perceptions of service quality. A total of 16 private oncology centres across Tamil Nadu were included in the study. The selection of oncology centres was based on accessibility and feasibility of obtaining institutional permission for data collection. As the study involved evaluation of service quality within healthcare institutions, obtaining approvals was a time-intensive process, and several hospitals were unwilling to participate due to concerns regarding institutional confidentiality and reputational sensitivity. Accordingly, a convenience sampling approach was adopted for the selection of oncology centres. Data collection was conducted only in centres that granted the necessary administrative approvals and ethical permissions for questionnaire administration. The participating centres permitted patients’ attendants to voluntarily respond to the survey.

To ensure adequate representation from each oncology centre and to satisfy the commonly accepted minimum sample size requirement for survey-based research and group-level statistical analysis, a minimum target of 30 responses per centre was established during the data collection process^28^. The overall sample size exceeded the minimum requirements for non-parametric testing and PLS-SEM, thereby ensuring adequate statistical power and stable model estimation. The selected cancer care facilities provided comprehensive services from diagnosis to palliative care. Participants represented diverse geographic regions, ranging from metropolitan to rural areas, thereby capturing variations in service delivery across different cancer care settings. Attendants aged 18 years and above, who were involved as either primary caregivers (patient’s day-to-day care) or secondary caregivers (providing supportive assistance), had accompanied patients for multiple hospital visits and were willing to provide informed consent were included in the study. Their repeated exposure to cancer care services enabled them to effectively assess service quality. Attendants who were unwilling to participate or below 18 years of age were excluded. To minimize potential response bias arising from predefined question order, the survey items were presented in a randomized sequence, as recommended by Youlin and Qian^29^.

### Questionnaire design

The survey instrument used in this study was developed through a structured and multi-stage process, combining established measurement approaches with context-specific adaptations relevant to oncology care. The study employs a performance-based measurement approach, drawing on the conceptual foundations of SERVPERF, to assess service performance perceptions without incorporating expectation-perception gaps. This approach allowed flexibility in capturing dimensions that are particularly relevant to cancer care settings. Item generation was guided by an extensive review of prior research and validated cancer care measuring instruments, including EORTC QLQ-C30 (European Organisation for Research and Treatment of Cancer Quality of Life Questionnaire Core 30—quality of life), FACT-G (Functional Assessment of Cancer Therapy—therapy outcomes), and CAHPS (Consumer Assessment of Healthcare Providers and Systems—patient experience). These instruments were not used as standalone scales, instead selected elements were adapted to inform the development of items related to patient experience, care processes, and outcome perceptions.

An initial pool of 38 service quality items was developed and subsequently refined through expert evaluation by four oncologists and ethics committee review, resulting in 30 items. A pilot study involving face-to-face interactions further improved clarity and contextual relevance. Based on expert input and participant feedback, additional context-specific aspects such as side-effect management, self-care support, privacy, service availability, and provider accessibility were incorporated.

Following iterative refinement, the final instrument consisted of 34 items, including 27 items measuring service quality and 7 items measuring attendant satisfaction. Service quality was conceptualized across six dimensions: tangibility, reliability of the process, reliability of services, responsiveness, assurance, and empathy. Attendant satisfaction was conceptualized as a comprehensive evaluation of the care experience, incorporating perceived value and behavioral intentions. The measurement constructs, dimensions, and corresponding items used in this study are presented in Table 1.

**Table 1:** Measurement constructs used in this study.

	Facet	Iems per construct	Explanation	Source
Service Quality Dimension	Tangibility	7	The availability and quality of physical facilities, medical equipment, and environment in cancer care services	EORTC QLQ-C30; Singh & Sidhu (2023) (Modified)
	Process Reliability	4	The ability to ensure consistent and dependable operational support within cancer care services	CAHPS (care coordination); Kamo et al. (2011) (Modified)
	Service Reliability	4	The ability to deliver cancer care services accurately, consistently, and as promised	CAHPS (timeliness); Gupta et al. (2013) (Modified)
	Responsiveness	4	The willingness to provide timely assistance and prompt support in cancer care services	SERVQUAL (Steele et al.2012);CAHPS (responsiveness); (Modified);Self-developed
	Assurance	3	The knowledge, competence, and courtesy of healthcare professionals, and their ability to instill trust and confidence in provision of cancer care services	FACT-G; Gupta et al. (2013) (Modified); Self-developed
	Empathy	5	Individualized attention and understanding of user needs are provided in in cancer care services	FACT-G; FAMCARE; Steele et al. (2012) (Modified); Self-developed
Customer Satisfaction	Expenses; quality; overall expectation	7	The overall evaluation of healthcare services based on perceived value for money, quality of care received, and the extent to which expectations are met	Kamo et al. (2011); Khatiban et al. (2019); CAHPS (Modified)

All constructs (see Table 5) were measured using a five-point Likert scale, ranging from 1 (strongly disagree/very poor) to 5 (strongly agree/excellent). The questionnaire also included demographic variables. A pilot study conducted with 30 respondents demonstrated acceptable internal consistency (Cronbach’s α ≥ 0.70), confirming the reliability of the instrument. The questionnaire was originally developed in English and translated into regional language (Tamil) to enhance participant comprehension. Ethical approval was obtained from the Institutional Ethics Committee and the Hospital Review Board, and informed consent was secured from all participants.

### Data analysis

Quantitative data were analyzed using IBM SPSS Statistics 22.0 and SmartPLS v.4.1.1.8. The threshold values for the following assessments were adopted based on established guidelines^30^. The adequacy of the dataset was initially assessed using the Kaiser–Meyer–Olkin (KMO) measure and Bartlett’s test of sphericity. The KMO value achieved from data of 0.793 exceeded the recommended threshold of 0.6 (Kaiser, 1974), indicating acceptable sampling adequacy. Bartlett’s test of sphericity was statistically significant (χ^2^ = 1332.332, df = 351, *p* < 0.001), confirming the suitability of the data for multivariate analysis.

Normality was assessed using the Kolmogorov–Smirnov and Shapiro–Wilk tests, as shown in Table 2. The results showed that all constructs significantly deviated from normality (*p* < 0.001), confirming the non-normal distribution of the data, which is consistent with the ordinal nature of Likert-scale responses. Due to the non-normal distribution of the data, non-parametric statistical tests were employed to examine differences across demographic groups. The Mann–Whitney U test was used for comparisons between two groups, while the Kruskal–Wallis test was applied for comparisons involving more than two groups. A significance level of *p* < 0.05 (two-tailed) was adopted for all analyses.

**Table 2:** Tests of normality.

	Kolmogorov-Smirnov^a^			Shapiro–Wilk		
	Statistic	df	Sig	Statistic	df	Sig
Tan	0.264	480	0.000	0.745	480	0.000
PR	0.264	480	0.000	0.774	480	0.000
SR	0.344	480	0.000	0.658	480	0.000
Resp	0.299	480	0.000	0.723	480	0.000
Ass	0.457	480	0.000	0.476	480	0.000
Emp	0.356	480	0.000	0.651	480	0.000
CS	0.364	480	0.000	0.663	480	0.000

^a^Lilliefors Significance Correction.

Effect sizes were calculated to assess the magnitude of differences. For the Mann–Whitney U test, the effect size (r) was interpreted using Cohen’s (1988) thresholds, where values of 0.10, 0.30, and 0.50 indicate small, medium, and large effects, respectively. Similarly, for the Kruskal–Wallis test, effect sizes (η^2^/ε^2^) were interpreted using thresholds of 0.01, 0.06, and 0.14, representing small, medium, and large effects, respectively^31^.

To assess the measurement and structural relationships, Partial Least Squares Structural Equation Modeling (PLS-SEM) was performed using SmartPLS v.4.1.1.8. The analysis included evaluation of construct reliability, convergent and discriminant validity, followed by evaluation of the structural model through path coefficients and their significance levels. In addition, an Importance-Performance Map Analysis (IPMA) was performed to provide deeper managerial insights by identifying the relative importance (total effects) and performance (average latent variable scores) of each service quality dimension in influencing attendant satisfaction. This analysis enabled the identification of priority areas for improvement by distinguishing constructs with high importance but relatively lower performance.

PLS-SEM was considered appropriate due to its robustness in handling non-normal data and its suitability for complex models. Overall, this analytical approach ensured a reliable evaluation of service quality perceptions while appropriately accounting for the characteristics of the data.

## Findings

### Inferential statistics

To examine whether service quality perceptions differed across demographic groups, appropriate non-parametric inferential statistical techniques were employed to test the proposed hypotheses, and the results are summarized in the following tables, with Table 3 presenting the demographic group comparisons.

**Table 3:** Statistical tests for demographic group comparisons. *Source*: Author’s Field Survey (2024 & 2025).

Category	Sample size n = 480	%	Mean Rank	U- Value	Z-value	*p*-value	Effect size (r)
1. Gender			Mann–Whitney U Test				
Male	230	48	260.82	*21,866.50*	− *3.890*	*0.0****00***	*0.178*
Female	250	52	212.25				
2. Age			Kruskal Wallis Test				
			Mean Rank	H	df	*p*-value	η2
21–30	100	21	180.96	*26.708*	*4*	***0.000***	*0.049*
31–40	94	20	223.27				
41–50	119	25	256.11				
51–60	118	25	251.46				
Above 60	49	10	282.47				
3. Education Level			Kruskal Wallis Test				
			Mean Rank	H	df	*p*-value	η2
Primary education	136	28	219.16	*11.273*	*3*	***0.010***	*0.017*
Secondary education	39	8	204.38				
Graduate	164	34	229.73				
Postgraduate	141	29	266.03				
4. Income Level			Kruskal Wallis Test				
			Mean Rank	H	df	*p*-value	η2
Below ₹10,000	16	3	227.25	3.483	5	0.626	0.000
₹10,000—₹30,000	15	3	288.85				
₹30,001—₹50,000	123	26	241.07				
₹50,001—₹1,00,000	76	16	231.21				
Above ₹1,00,000	39	8	251.69				
No Income	211	44	228.24				
5. Occupation			Kruskal Wallis Test				
			Mean Rank	H	df	*p*-value	η2
Government sector	15	3	233.19	11.908	6	0.064	0.012
Private sector	143	30	251.07				
Self—employed	51	11	199.15				
Homemaker	173	36	224.15				
Retired	57	12	230.16				
Student	21	4	293.00				
Unemployed	20	4	273.47				
6. Relationship to the Patient			Kruskal Wallis Test				
			Mean Rank	H	df	*p*-value	η2
Spouse	221	46	252.92	*62.832*	*6*	***0.000***	*0.120*
Son/Daughter	151	31	198.67				
Siblings	39	8	195.59				
Parent	28	6	340.11				
Son/ D/o- in law	18	4	431				
Grand—son/ D/o	19	4	139.61				
Care taker	4	1	375.50				
7. Primary Attendant of the Patient			Mann–Whitney U Test				
			Mean Rank	U- Value	Z-value	*p*-value	r
Yes	418	87	244.42	11,262.0	− 0.531	0.595	0.024
No	62	13	234.27				
8. Financing Categories			Kruskal Wallis Test				
			Mean Rank	H	df	*p*-value	η2
Government Insurance	231	47	192.24	*52.854*	*5*	***0.000***	*0.098*
Private Insurance	79	16	268.90				
Paid service	75	15	289.55				
Schemes (ESI/ECHS)	70	14	286.12				
Government Insurance & Paid	36	7	241.36				
Private Insurance & Paid	4	1	166.63				
9. Number of hospital visits in the past year			Kruskal Wallis Test				
			Mean Rank	H	df	*p*-value	η2
Less than 10 times	163	34	276.90	*32.601*	*5*	***0.000***	*0.058*
11 to 20 times	82	17	225.46				
21 to 30 times	59	12	211.18				
31–40 times	54	11	223.27				
41–50 times	57	12	241.25				
More than 50	65	14	170.74				
10. Residential Status			Kruskal Wallis Test				
			Mean Rank	H	df	*p*-value	η2
Urban	288	60	244.65	*17.749*	*2*	***0.000***	*0.033*
Semi-urban	91	19	235.96				
Rural	101	21	144.99				

The results indicated that gender showed a statistically significant difference in service quality (U = 21,866.500, Z = − 3.890, *p* < 0.001, r = 0.178), with a small to moderate effect size, where male respondents (Mean Rank = 260.82) reported higher service quality than females. Similarly, age demonstrated significant differences (H = 26.708, df = 4, *p* < 0.001, η^2^ = 0.049), indicating a small effect, with respondents aged above 60 years (Mean Rank = 282.47) reporting higher service quality. Educational level also showed significant variation (H = 11.273, df = 3, *p* = 0.010, η^2^ = 0.017), with a small effect size, where postgraduates (Mean Rank = 266.03) reported higher service quality. In contrast, income level did not exhibit significant differences (H = 3.483, df = 5, *p* = 0.626, η^2^ ≈ 0.000), indicating a negligible effect. Likewise, occupation was not statistically significant (H = 11.908, df = 6, *p* = 0.064, η^2^ = 0.012), reflecting a small effect. However, the number of hospital visits showed significant differences in service quality (H = 32.601, df = 5, *p* < 0.001, η^2^ = 0.058), indicating a small to moderate effect, where respondents with less than 10 visits (Mean Rank = 276.90) reported higher service quality. A strong significant difference was observed for relationship to the patient (H = 62.832, df = 6, *p* < 0.001, η^2^ = 0.120), with a moderate effect size, where son/daughter-in-law (Mean Rank = 431.00) reported the highest service quality. In contrast, primary caregiver status did not show a significant difference (U = 11,262.000, Z = − 0.531, *p* = 0.595, r = 0.024), indicating a negligible effect. Furthermore, financing category demonstrated a significant difference (H = 52.854, df = 5, *p* < 0.001, η^2^ = 0.098), indicating a moderate effect, where paid service users (Mean Rank = 289.55) reported higher service quality. Finally, residential status also showed significant variation (H = 17.749, df = 2, *p* < 0.001, η^2^ = 0.033),

with a small effect size, where urban respondents (Mean Rank = 244.65) reported higher service quality than others. Overall, the findings suggest that while several demographic variables significantly influence service quality, their practical impact ranges from negligible to moderate. Since significant differences were observed across certain demographic groups, a structural analysis was required to understand which service dimensions most strongly contribute to the attendant satisfaction.

### Collinearity assessment

Prior to conducting the SEM analysis, multicollinearity was examined to ensure that the data met the assumptions required for multiple regression analysis^32^. The Variance Inflation Factor (VIF) was employed to assess the extent of collinearity among the indicators. As presented in Table 4, all VIF values were found to be well below the recommended threshold of 10.0^33^, indicating the absence of multicollinearity issues in the dataset. In addition, the study assessed the presence of common method variance (CMV) using Harman’s single-factor test in IBM SPSS Statistics. The results revealed that the first factor accounted for 30.42% of the total variance, which is below the critical threshold of 50%.^34^. This suggests that CMV does not pose a significant threat to the validity of the findings, thereby confirming the suitability of the data for further analysis.

**Table 4:** Reliability, Validity and multi-collinearity of the construct. *Source*: Author’s Field Survey Analysis (2024 & 2025)

Construct	Item	Outer loading	CR	AVE	Cronbach Alpha	VIF
Service quality dimensions						
1. Tangibility (7 items)						
T1	The hospital is easy to reach, with good parking and transport options	0.627	0.869	0.537	0.821	1.383
T2	The hospital is clean and well-maintained	0.769				1.835
T3	The waiting areas are spacious and comfortable for patients and attendants	0.918				4.019
T4	Navigation and direction to different departments within the hospital are easy	0.888				3.452
T5	Hospital’s accessibility features, such as ramps, elevators, and wheelchair facilities, are adequate	0.610				1.408
T6	The room service provided during my stay was satisfactory	0.486				1.205
T7	Medical equipment I interact with or oversee is in good and working condition	0.162				–
2. Process Reliability (4 items)						
PR 1	Staff members from different departments coordinate well with each other for patient care	0.839	0.902	0.755	0.838	1.689
PR 2	The hospital admission process for patients is effective and efficient	0.260				–
PR 3	The hospital makes the discharge process for patients easy and quick	0.889				2.236
PR 4	Billing processes are managed efficiently	0.878				2.256
3. Service Reliability (4 items)						
SR 1	Appointment scheduling for patients is timely	0.840	0.837	0.567	0.746	1.646
SR 2	Consistency of the patient’s appointment and treatment schedules is satisfactory	0.666				1.302
SR 3	Test results reach patients promptly and easily	0.848				1.700
SR 4	Required medications are readily available at the pharmacy for patient care	0.632				1.284
4. Responsiveness (4 items)						
RESP 1	Doctors are accessible for consultations with minimal waiting time	0.867	0.910	0.716	0.868	2.214
RESP 2	Assistance provided to the patient in managing pain, sleep issues, and anxiety is adequate	0.841				2.202
RESP 3	Contacting hospital staff for urgent questions or emergencies related to the patient is easy	0.825				1.893
RESP 4	Responses to the questions or concerns raised by the patient during their treatment are prompt	0.852				2.187
5.Assurance (3 items)						
ASS 1	Doctors communicate the process of diagnosis, treatment, and prognosis effectively and clearly	0.837	0.821	0.608	0.708	1.632
ASS 2	The patient’s privacy is maintained during examinations and treatments	0.646				1.485
ASS 3	Hospital staff consistently follow safety procedures and hygienic practices, including the use of gloves, PPE in designated facilities	0.841				1.258
6. Empathy (5 items)						
EMP 1	The healthcare team effectively educates patients about potential side effects of treatment	0.883	0.873	0.585	0.815	2.577
EMP 2	Doctors offer effective guidance on health maintenance and self-care for the patient after treatment	0.589				1.424
EMP 3	Healthcare providers handle the patient’s care with professionalism and courtesy	0.826				2.145
EMP 4	Healthcare providers show care and compassion	0.601				1.400
EMP 5	Clerks and receptionists are helpful, courteous, and respectful	0.870				2.311
Attendant satisfaction						
Satisfaction (7 items)						
SAT 1	The quality of care provided reflects good value	0.837	0.936	0.679	0.917	2.416
SAT 2	The services provided meet expectations for quality of care	0.803				2.718
SAT 3	Financial assistance options, including insurance coverage and payment plans, are effective	0.572				1.479
SAT 4	Healthcare services address the needs and preferences of the patient	0.899				3.987
SAT 5	The hospital is recommendable to others based on the overall value of care received	0.878				5.585
SAT 6	Continuity of care with this healthcare provider is preferable	0.923				6.350
SAT 7	The overall quality of care provided throughout treatment is satisfactory	0.803				1.926

n = 480

### Measurement model

The measurement model was assessed to evaluate the adequacy and quality of the study constructs. This involved examining indicator reliability, internal consistency reliability, and convergent validity. The results showed that all items loaded significantly on their respective constructs, with outer loadings ranging from 0.604 to 0.949. Although loadings above 0.70 are generally recommended, values between 0.40 and 0.70 are considered acceptable when supported by adequate Average Variance Extracted (AVE > 0.50) and Composite Reliability (CR > 0.70), as threshold values were assessed based on recommended criteria^35^. Accordingly, items T7 and PR2, which had loadings below 0.40, were removed from further analysis. Internal consistency reliability was assessed using Cronbach’s alpha and Composite Reliability. Cronbach’s alpha values ranged from 0.708 to 0.917, while Composite Reliability values ranged from 0.811 to 0.936, exceeding the recommended threshold of 0.70. These results confirm that the constructs demonstrate satisfactory reliability. Convergent validity was evaluated using the Average Variance Extracted (AVE). All constructs reported AVE values above the threshold of 0.50, indicating that the constructs explain a substantial proportion of variance in their indicators. Therefore, the measurement model demonstrates adequate convergent validity (see Table 4).

Discriminant validity was assessed using the Fornell–Larcker criterion (Fornell & Larcker, 1981). As presented in Table 5, the square root of the Average Variance Extracted (AVE) for each construct was greater than its correlations with other constructs in the model. This indicates that each construct shares more variance with its own indicators than with other constructs. All latent constructs satisfied the recommended criteria^33^, thereby confirming that discriminant validity is adequately established in this study.

**Table 5:** Discriminant Validity as per Fornell-Larker criterion.

	ASS	EMP	CS	PR	RESP	SR	TAN
ASS	**0.771**						
EMP	0.549	**0.766**					
CS	0.315	0.740	**0.906**				
PR	0.567	0.677	0.714	**0.869**			
RESP	0.579	0.694	0.804	0.817	**0.847**		
SR	0.521	0.724	0.697	0.687	0.765	**0.853**	
TAN	0.543	0.641	0.795	0.760	0.840	0.739	**0.844**

### Structural model

**Figure 2:**
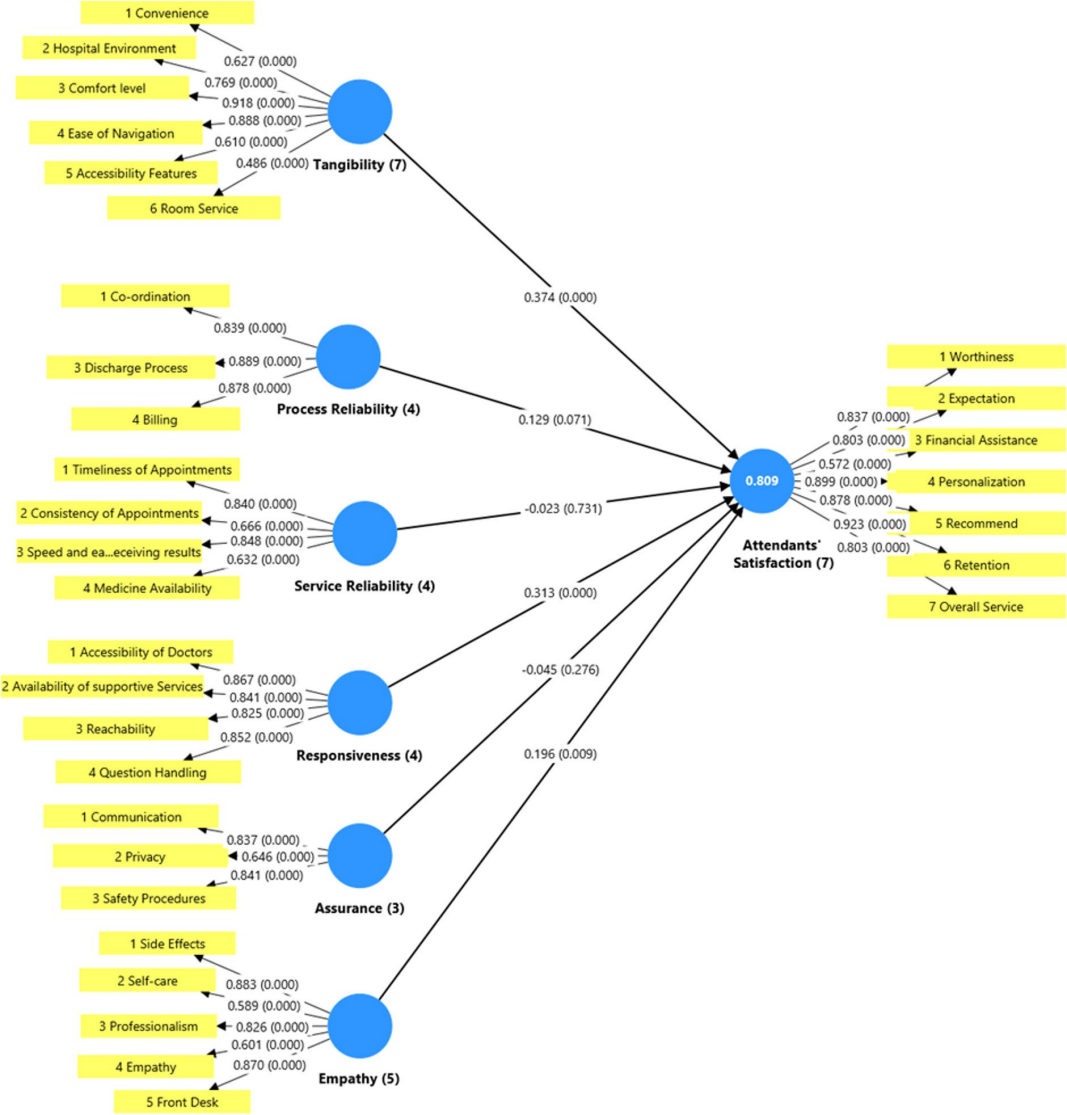
Path analysis showing predictors of attendants’ satisfaction.

The structural model was assessed to examine the hypothesized relationships among the constructs and to evaluate the model’s predictive capability. Path coefficients were estimated using a bootstrapping procedure with 5,000 subsamples, following recommended guidelines^36^. The significance of the structural paths was determined using p-values. In addition, the model’s explanatory power was evaluated using the coefficient of determination (R^2^), which reflects the extent to which the endogenous construct (DV) is explained by the exogenous variables (IV). The results of the structural model are presented in Table 6, while the overall model with hypothesized relationships is illustrated in Fig. 2.

**Table 6:** Hypothesis Testing (Structural model). *Source*: Survey Results.

Hypothesis	β—value	CI		t-value	Effect size		*p*-value	Decision
		2.5%	97.5%		f^2^	effect		
H1: Tan → CS	0.374	0.230	0.488	5.678	0.107	Small	**0.000**	Supported
H2: PR → CS	0.129	0.006	0.286	1.805	0.113	Small	0.071	Rejected
H3: SR → CS	− 0.023	− 0.155	0.107	0.344	0.002	Negligible	*0.731*	Rejected
H4: RESP → CS	0.313	0.169	0.449	4.403	0.203	Medium	**0.000**	Supported
H5: ASS → CS	− 0.045	− 0.129	0.031	1.090	0.008	Negligible	*0.276*	Rejected
H6: EMP → CS	0.196	0.046	0.345	2.606	0.045	Small	**0.009**	Supported
R^2^	0.809	≥ 0.67 (Substantial)						
Predictive Relevance (Q2)	0.793	Q2 > 0						
Estimated model fit indices (SRMR)	0.083							
The values of f^2^: 0.02 = small, 0.15 = medium, 0.35 = large

The structural model results indicate that tangibility (β = 0.374, *p* < 0.001), responsiveness (β = 0.313, *p* < 0.001), and empathy (β = 0.196, *p* = 0.009) have a positive and statistically significant influence on customer satisfaction, thereby supporting H1, H4, and H6. Among these, tangibility and responsiveness emerge as the strongest predictors, with responsiveness demonstrating a moderate effect size (f^2^ = 0.203), while tangibility (f^2^ = 0.107) and empathy (f^2^ = 0.045) exhibit small effect sizes. In contrast, process reliability (β = 0.129, *p* = 0.071), service reliability (β = − 0.023, *p* = 0.731), and assurance (β = − 0.045, *p* = 0.276) do not show a statistically significant impact on customer satisfaction, leading to the rejection of H2, H3, and H5. The effect sizes for these constructs are negligible to small, indicating limited practical influence on satisfaction in this context.

Overall, the customer satisfaction is primarily driven by tangible aspects of service, prompt responsiveness, and empathetic interactions.

The coefficient of determination (R^2^) for Customer Satisfaction (CS) was found to be 0.809, indicating that approximately 80.9% of the variance in CS is explained by the exogenous constructs. According to established guidelines, this represents substantial explanatory power, suggesting that the model provides a strong explanation of attendant satisfaction in cancer care^37^.

To assess predictive performance, the PLS predict procedure was employed. The Q^2^predict value for CS was 0.793, which is significantly greater than zero, indicating strong out-of-sample predictive relevance. The close correspondence between R^2^ (0.809) and Q^2^predict (0.793) confirms that the model is not overfitted and exhibits strong generalizability. The SRMR (standardized root mean square residual) was found to be 0.083 (see Table 6). As a model fit index, SRMR values below 0.10 are generally considered indicative of a satisfactory model fit^36^.

### IPMA (importance—performance map analysis)

While PLS-SEM identifies the significant predictors of attendant satisfaction, Importance—Performance Map Analysis (IPMA) provides deeper insights by evaluating each construct based on its importance (total effect on satisfaction) and performance. This approach enables a more practical interpretation of the results by highlighting areas that require managerial attention^38^. The IPMA results reveal that service quality dimensions do not contribute equally to attendant satisfaction. Constructs demonstrating high importance but comparatively lower performance emerge as critical areas requiring managerial intervention and prioritised resource allocation. In contrast, constructs with lower importance have a limited influence on satisfaction, regardless of their performance levels, and are therefore considered lower priority.

**Figure 3:**
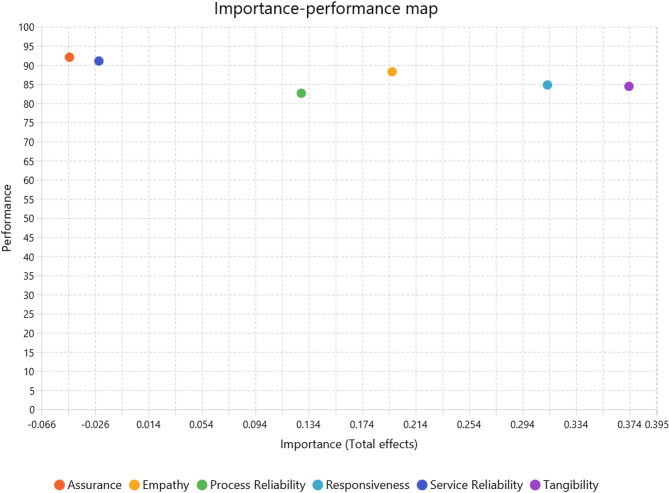
IPMA—importance-performance map analysis.

In the present study from Fig. 3 it is evident that, tangibility (importance = 0.374, performance ≈85) and responsiveness (importance = 0.313, performance ≈85) emerge as the most influential determinants of attendant satisfaction. However, since their performance is already at a good level, they are considered areas to be maintained rather than immediately improved. Empathy (importance = 0.196, performance ≈88), although demonstrating slightly higher performance, still holds considerable importance, indicating that further enhancements in interpersonal care and emotional support can improve satisfaction and making it a key area for targeted refinement. Process reliability (importance = 0.129, performance ≈83), with comparatively lower performance and moderate importance, can be considered a secondary area for improvement, however its effect is only marginally significant at 10% level, suggesting that the impact of improvements may be limited. In contrast, assurance and service reliability, despite exhibiting excellent performance levels above 90, show negligible importance and are therefore interpreted as basic factors that require maintenance but not additional investment.

## Discussion and implications

### Discussion

This study examined the determinants of service quality and their influence on attendant satisfaction in cancer care settings, along with demographic variations in perception. The findings demonstrate that the service quality perceptions differed significantly across demographic groups such as gender, age, education, residential status, financing category, and frequency of hospital visits. These findings indicate that personal characteristics and prior exposure to healthcare systems play an important role in shaping how attendants interpret and assess service encounters, consistent with evidence that socio-demographic factors significantly influence perceptions of care quality^39^. Educated and urban residents consistently reported higher satisfaction, likely due to greater familiarity with healthcare systems, enhanced health literacy, or lower expectation thresholds. This pattern aligns with studies showing that individuals with higher education and urban backgrounds typically report better healthcare experiences due to greater system awareness and stronger health literacy^40,41^. Additionally, research has documented that older adults and males often express higher satisfaction because they tend to have more stable expectations and greater trust in healthcare providers^42,43^. Attendants with fewer hospital visits also reported more positive perceptions, which may reflect reduced exposure to system inefficiencies compared to frequent visitors. Similar findings have been observed in studies showing that frequent users of healthcare services are more likely to encounter delays, inconsistent processes, or unmet expectations, leading to lower satisfaction^44,45^. The inferential analysis revealed that several demographic factors significantly influence service quality perceptions; however, their effect sizes were predominantly small to moderate, indicating limited practical impact. This suggests that while demographic characteristics shape perceptions to some extent, service-related factors play a more critical role in determining satisfaction, which is supported by a study by Pompili et al.^46^, where treatment decision efficacy a service-related factor was identified as the sole significant predictor of satisfaction after adjusting for demographic variables, highlighting the critical importance of care delivery processes over patient characteristics.

The structural model’s identification of tangibility, responsiveness, and empathy as significant predictors of attendant satisfaction aligns with recent evidence that hospital service quality remains strongly associated with patient and Attendant satisfaction under modern care settings^47^. Tangibility emerged as the strongest predictor, underscoring the importance of physical facilities, ease of navigation, and environmental comfort in easing attendant stress, while responsiveness and empathy highlight the critical role of timely assistance, staff attentiveness, and compassionate communication.

In contrast, process reliability, service reliability, and assurance showed non-significant effects on satisfaction. This result is inconsistent with several studies in the literature review that report a positive relationship between these dimensions and satisfaction. However, not all research led to similar conclusions. A plausible explanation is that these dimensions may function as a baseline factors, where their presence is expected and does not necessarily enhance satisfaction unless they are inadequate. Supporting this view, a study by Paul^48^ also indicate that certain process-related aspects may not directly influence satisfaction but instead contribute indirectly through improved efficiency and care delivery. This explains why reliability-related dimensions, despite being essential, did not emerge as key differentiators in this study.

The IPMA results further strengthen these findings by providing actionable insights. Empathy, however, emerges as a priority area for improvement, as it combines high importance with potential for enhancement. This finding is supported from a study by Launonen et al.^49^ emphasizes that empathetic communication and emotional support significantly influence both patient and caregiver satisfaction. Studies have shown that caregivers often experience psychological stress and uncertainty during cancer treatment, and their satisfaction is closely linked to how well healthcare providers address their emotional and informational needs^50^. Collectively, these findings emphasize the need for hospitals to strengthen expectations of today’s oncology Attendants.

### Managerial implications

The study offers important implications for oncology practitioners and administrators. Responsiveness emerged as a significant determinant of attendant satisfaction, emphasizing the importance of reducing waiting times, ensuring timely communication, and improving accessibility to healthcare professionals. In oncology settings, where treatment processes are prolonged and emotionally demanding, attendants frequently depend on continuous interaction with healthcare providers for information, reassurance, and care coordination^51^. Previous studies have similarly highlighted the importance of communication and accessibility in influencing caregiver experiences and perceived quality of care^52,53^. The findings also highlight the continued importance of tangible service attributes, including hospital infrastructure, cleanliness, comfort, and the overall care environment. Although tangible dimensions may not directly influence clinical outcomes, they contribute substantially to perceptions of safety, trust, and professionalism during extended treatment processes^6^. Prior research in oncology care has similarly reported that supportive physical environments positively influence patient and caregiver experiences in long-term treatment settings^54,55^. In addition, empathy emerged as an important dimension associated with attendant satisfaction, underscoring the need for caregiver-centered communication and emotionally supportive care practices. Oncology attendants often experience substantial psychological and emotional burden while supporting patients throughout diagnosis, treatment, and recovery^56^. Accordingly, the findings reinforce the importance of training healthcare professionals in empathetic communication, emotional support, and inclusive care interactions. Existing studies similarly indicate that caregiver satisfaction is strongly associated with information clarity, emotional reassurance, and involvement in treatment-related decision-making^57,58^. Overall, the findings suggest that oncology healthcare administrators should prioritize high-relevance service dimensions while maintaining baseline standards may support improved attendant experience.

### Theoretical implications

This study contributes to the healthcare service quality literature by demonstrating that not all service quality dimensions are equally associated with satisfaction in oncology settings. The findings highlight the need for context-specific adaptation of service quality models, particularly in complex and emotionally intensive healthcare environments. The study further extends existing literature by incorporating the perspectives of patient attendants, a stakeholder group that remains comparatively underexplored in oncology service quality research despite their significant involvement in caregiving and treatment navigation. By focusing on attendant perceptions, the study broadens the conceptual understanding of healthcare service quality beyond patient-centered evaluations and highlights the importance of attendant service experiences in oncology settings. The study further extends Service-Dominant Logic (SDL) theory by emphasizing the importance of relational and interaction-based aspects of healthcare service delivery in shaping satisfaction outcomes. In oncology settings, satisfaction appears to be shaped not only by technical service delivery but also by continuous interactions among healthcare providers, patients, and attendants^59^. The results indicate that communication, emotional support, and empathetic care practices exert greater influence on satisfaction than operational efficiency alone. Similar observations have been reported by Nembhard et al.^60^ and Wang et al.^61^, who identified relational and emotional dimensions as important determinants of caregiver and patient experiences. Furthermore, the results support the applicability of performance-based service quality assessment approaches in healthcare settings. Rather than emphasizing expectation-perception gaps, the findings suggest that actual service experiences provide a more contextually relevant understanding of satisfaction in prolonged care environments such as oncology. Contemporary oncology care studies suggest that experience-based evaluations provide more accurate insights into satisfaction, particularly in long-term care contexts^62,63^. Overall, the study advances the theoretical understanding of oncology service quality by emphasizing the significance of experiential, relational, and caregiver-oriented dimensions in shaping attendant satisfaction.

## Conclusion

In conclusion, this study provides a comprehensive understanding of attendants’ perceptions of service quality in cancer care, along with insights into how different service dimensions are associated with their satisfaction. The findings indicate that tangibility, responsiveness, and empathy are more strongly associated with attendant satisfaction, while reliability and assurance show comparatively limited association within the study context. Empathy emerges as an area where attendants report higher expectations, highlighting the relevance of emotional support and patient-centered interactions in oncology settings. The analysis also reveals notable demographic variations, suggesting that attendants’ expectations and experiences differ based on factors such as age, gender, education, and prior healthcare exposure. This suggests that Attendant experiences are not uniform and should be addressed through tailored service approaches that consider these differences. While the results contribute valuable insights for enhancing the attendants experience, the study is limited by its regional focus, reliance on self-reported perceptions, and cross-sectional design, which may restrict generalizability and overlook changes in satisfaction over time. The study also leaves scope for exploring other organizational and clinical factors that may be associated with attendant experiences. Future research could eliminate these limitations by employing longitudinal approaches, expanding to broader healthcare contexts, and incorporating additional variables to give a more holistic picture of service quality in cancer care.

## Data Availability

In compliance with privacy protocols and confidentiality agreements, the dataset from this study is not accessible to the public but are available from the corresponding author on reasonable request.

## References

[1] Leung, K. et al. Quality indicators for systemic anticancer therapy services: A systematic review of metrics used to compare quality across healthcare facilities. *Eur. J. Cancer.* **195**, 113389 (2023). 37924649 10.1016/j.ejca.2023.113389 PMC10697827

[2] Bhadelia, A. Comprehensive value-based cancer care in India. *Indian J. Med. Res.* **154**, 329–337 (2021). 35142652 10.4103/ijmr.IJMR_4251_20 PMC9131774

[3] Chintapally, N. et al. State of cancer care in India and opportunities for innovation. *Future Oncol.* **19**, 2593–2606 (2023). 37675499 10.2217/fon-2023-0047

[4] Pramesh, C. S. et al. Delivery of affordable and equitable cancer care in India. *Lancet Oncol.* **15**, e223–e233 (2014). 24731888 10.1016/S1470-2045(14)70117-2

[5] Shulman, L. N. Cancer care quality: Current state and future directions. *J. Clin. Oncol. Educ. Book.* **35**, e337–e341 (2015). 10.14694/EdBook_AM.2015.35.e337 25993194

[6] Gupta, D., Markman, M., Rodeghier, M. & Lis, C. G. The relationship between patient satisfaction with service quality and survival in pancreatic cancer. *Patient Prefer Adherence.* **6**, 765–772 (2012). 23152670 10.2147/PPA.S37900 PMC3496532

[7] Kittang, J., Ohlsson-Nevo, E. & Schröder, A. Quality of care in the oncological outpatient setting: Individual interviews with people receiving cancer treatment. *Eur. J. Oncol. Nurs.* **64**, 102335 (2023). 37290164 10.1016/j.ejon.2023.102335

[8] Zarei, E. Service quality of hospital outpatient departments: Patients’ perspective. *Int. J. Health Care Qual. Assur.* **28**, 778–790 (2015). 26440482 10.1108/IJHCQA-09-2014-0097

[9] Mahran, S. & Nagshabandi, E. Oncology patients’ satisfaction towards quality health care services at accredited university hospital. *Am. J. Nurs. Sci.* **5**, 162–168 (2016).

[10] Applebaum, A. J. & Breitbart, W. Care for the cancer caregiver: A systematic review. *Palliat. Supp. Care.* **11**, 231–252 (2013). 10.1017/S1478951512000594 PMC4973511 23046977

[11] Dionne-Odom, J. N. et al. Family caregiver roles and challenges in assisting patients with cancer treatment decision-making: Analysis of data from a national survey. *Health Expect.* **26**, 1965–1976 (2023). 37394734 10.1111/hex.13805 PMC10485321

[12] Hazelwood, D. M., Koeck, S., Wallner, M., Anderson, K. H. & Mayer, H. Patients with cancer and family caregivers: Management of symptoms caused by cancer or cancer therapy at home. *HeilberufeSCIENCE* **3**, 149–158 (2012). 24027658 10.1007/s16024-012-0118-z PMC3767444

[13] Nila, S., Dutta, E., Prakash, S. S., Korula, S. & Oommen, A. M. Patient and caregiver perspectives of select non-communicable diseases in India: A scoping review. *PLoS ONE* **19**, e0296643 (2024). 38180969 10.1371/journal.pone.0296643 PMC10769076

[14] Braimah, J. A. et al. Effects of demographic and socio-economic factors on dissatisfaction with formal healthcare utilisation among older adults with very low incomes in Ghana. *Cogent Public Health.* **9**, 2108568 (2022).

[15] Mini, M., Sabu, K. M. & Kuriakose, J. Perceived healthcare quality as the predictor of patient satisfaction: Findings from a public sector tertiary care hospital in Kerala, South India. *Clin. Epidemiol. Glob. Health.* **25**, 101563 (2025).

[16] Devaraju. Patient satisfaction of health service delivery in primary health centres: A socio-demographic study. *Int. J. Res. Anal. Rev.* **12**:245–252 (2025).

[17] Batta, G., Mundhe, D. K., Vishnuprasad, R., Batta, A. & Baruah, M. M. Demographic determinants of patient satisfaction in medical outpatient department of a tertiary care hospital in North India. *J. Family Med. Prim. Care.* **14**, 3469–3473 (2025). 41041186 10.4103/jfmpc.jfmpc_430_25 PMC12488123

[18] Rahmatia, S. et al. Service quality in hospital inpatient care: SERVQUAL model approach. *Health SA Gesondheid.* **30**, 3055 (2025). 41069560 10.4102/hsag.v30i0.3055 PMC12505488

[19] Rauf, A., Muhammad, N., Mahmood, H. & Yen, Y. Y. The influence of healthcare service quality on patients’ satisfaction in urban areas: The case of Pakistan. *Heliyon.* **10**, e37506 (2024). 39323768 10.1016/j.heliyon.2024.e37506 PMC11422050

[20] Hosseinzadeh, M., Pouladzadeh, M. & Eskandari, A. Assessment of healthcare service quality and patient satisfaction using the SERVQUAL questionnaire in Khuzestan Province during 2022–2023. *Jundishapur. J. Chronic. Dis. Care.* **13**, e146329 (2024).

[21] Bin Libdah, S. M. & Alqurashi, M. A. Impact of health service quality on patient loyalty in government and private hospitals in the Asir region, Saudi Arabia. *Cureus.* **18**, e100963 (2026). 41658728 10.7759/cureus.100963 PMC12877410

[22] Ali, J., Jusoh, A., Idris, N. & Nor, K. M. Healthcare service quality and patient satisfaction: A conceptual framework. *Int. J. Qual. Reliab. Manag.*10.1108/ijqrm-04-2022-0136 (2023).

[23] Alfatafta, M. et al. Assessing service quality and its impact on patient experience and satisfaction in prosthetics and orthotics: A SERVQUAL-based cross-sectional study. *BMC Health Serv. Res.* **25**, 985 (2025). 40721773 10.1186/s12913-025-13172-z PMC12306002

[24] Yunus, N. M., Abdullah, M. Z., Binti Ramdan, N. F. & Alnuaimi, H. A. S. B. S. The impact of healthcare service quality on patient satisfaction at university health center. *Inf. Manag. Bus. Rev.* **16**, 440–451 (2024).

[25] Al-Awadi, A. A. A., Chabchoub, I. & Falah, M. A. Cancer patients satisfaction and quality of healthcare services in Iraq: A cross-sectional study to evaluate the quality of care in cancer management. *Asian Pac. J. Cancer Prev.* **25**, 2159–2167 (2024). 38918679 10.31557/APJCP.2024.25.6.2159 PMC11382871

[26] Vastardi, M., Govina, O., Kalokairinou, A., Tsamasiotis, G. K. & Kalemikerakis, I. Cancer patients’ satisfaction with day care unit services have impact on their quality of life. *Med. Pharm. Rep.* **98**, 486–496 (2025). 41221459 10.15386/mpr-2846 PMC12600069

[27] Gupta, D., Rodeghier, M. & Lis, C. G. Patient satisfaction with service quality in an oncology setting: Implications for prognosis in non-small cell lung cancer. *Int. J. Qual. Health Care.* **25**(6), 696–703 (2013). 24123242 10.1093/intqhc/mzt070 PMC3842127

[28] Memon, M. A. et al. Sample size for survey research: Review and recommendations. *J. Appl. Struct. Equ. Model.* **4**(2), i–xx (2020).

[29] Youlin, H. & Qian, L. Understanding the potential adoption for autonomous vehicles in China: The perspective of behavioral reasoning theory. *Psychol. Mark.* **38**, 7–73 (2021).

[30] Li, N., Huang, J. & Feng, Y. Construction and confirmatory factor analysis of the core cognitive ability index system of ship C2 system operators. *PLoS ONE* **15**, e0237339 (2020). 32833969 10.1371/journal.pone.0237339 PMC7446803

[31] Peres, F. F. Effect sizes for nonparametric tests. *Biochem. Med.* **36**, 010101 (2026). 10.11613/BM.2026.010101 PMC12701665 41399660

[32] Hair, J. F., Black, W. C., Babin, B. J. & Anderson, R. E. *Multivariate Data Analysis* 7th edn. (Pearson, 2010).

[33] Hair, J. et al. *A Primer on Partial Least Squares Structural Equation Modeling (PLS-SEM)* 2nd edn. (Sage, 2017).

[34] Jia, H. et al. A new perspective for improving the human resource development of primary medical and health care institutions: A structural equation model study. *Int. J. Environ. Res. Public Health.* **18**, 2560 (2021). 33806526 10.3390/ijerph18052560 PMC7967509

[35] Chinyamurindi, W., Mathibe, M. & Hove-Sibanda, P. Social enterprise performance in South Africa: The role of strategic planning and networking capability. *J. Soc. Entrep.* **16**, 771–788 (2025).

[36] Henseler, J., Hubona, G. & Ray, P. A. Using PLS path modeling in new technology research: Updated guidelines. *Ind. Manag. Data Syst.* **116**, 2–20 (2016).

[37] Chicco, D., Warrens, M. J. & Jurman, G. The coefficient of determination R-squared is more informative than SMAPE, MAE, MAPE, MSE and RMSE in regression analysis evaluation. *PeerJ Comput. Sci.* **7**, e623 (2021). 34307865 10.7717/peerj-cs.623 PMC8279135

[38] García-Fernández, J. et al. Importance-performance matrix analysis (IPMA) to evaluate servicescape fitness consumer by gender and age. *Int. J. Environ. Res. Public Health.* **17**, 6562 (2020). 32916936 10.3390/ijerph17186562 PMC7557594

[39] Manulik, S., Karniej, P. & Rosińczu, J. The influence of socio-demographic characteristics on respondents’ perceptions of healthcare service quality. *J. Educ. Health Sport.* **8**, 708–724 (2018).

[40] Al-Abri, R. & Al-Balushi, A. Patient satisfaction survey as a tool towards quality improvement. *Oman Med J.* **29**, 3–7 (2014). 24501659 10.5001/omj.2014.02 PMC3910415

[41] Prakash, B. Patient satisfaction. *J. Cutan. Aesthet. Surg.* **3**, 151 (2010). 21430827 10.4103/0974-2077.74491 PMC3047732

[42] Rahmqvist, M. & Bara, A. C. Patient characteristics and quality dimensions related to patient satisfaction. *Int. J. Qual. Health Care.* **22**, 86–92 (2010). 20133477 10.1093/intqhc/mzq009

[43] Bleich, S. How does satisfaction with the health-care system relate to patient experience?. *Bull. World Health Organ.* **87**, 271–278 (2009). 19551235 10.2471/BLT.07.050401 PMC2672587

[44] Mularczyk-Tomczewska, P., Gujski, M., Koperdowska, J. M., Wójcik, J. & Silczuk, A. Factors influencing patient satisfaction with healthcare services in Poland. *Med. Sci. Monit.* **31**, e948225 (2025). 40534114 10.12659/MSM.948225 PMC12186550

[45] Xesfingi, S. & Vozikis, A. Patient satisfaction with the healthcare system: Assessing the impact of socio-economic and healthcare provision factors. *BMC Health Serv. Res.* **16**, 94 (2016). 26979458 10.1186/s12913-016-1327-4 PMC4793546

[46] Pompili, C. et al. Factors influencing patient satisfaction after treatments for early-stage non-small cell lung cancer. *J. Cancer Res. Clin. Oncol* **148**, 2447–2454 (2022). 34515847 10.1007/s00432-021-03795-0 PMC9349300

[47] Rahmatia, S. et al. Service quality in hospital inpatient care: SERVQUAL model approach. *Health SA Gesondheid*10.4102/HSAG.v30i0.3055 (2025). 41069560 10.4102/hsag.v30i0.3055 PMC12505488

[48] Paul. J. Healthcare leadership: How do we maximize patient satisfaction and loyalty in healthcare facilities? *Med. Res. Arch.* **13** (2025).

[49] Launonen, M., Vehviläinen-Julkunen, K., Mikkonen, S. & Kvist, T. Care quality and satisfaction at the cancer hospital: A questionnaire study of older patients with cancer and their family members. *BMC Health Serv. Res.* **24**, 190 (2024). 38342900 10.1186/s12913-024-10646-4 PMC10860216

[50] Zeng, Z., Holtmaat, K. & Koole, S. L. Psychological care for cancer survivors: A 2 × 2 model of interpersonal emotion regulation by caregivers. *Front Psychol.* **15**, 1390692 (2024). 38979076 10.3389/fpsyg.2024.1390692 PMC11228138

[51] DuBenske, L. L., Chih, M. Y., Gustafson, D. H., Dinauer, S. & Cleary, J. F. Caregivers’ participation in the oncology clinic visit mediates the relationship between their information competence and their need fulfillment and clinic visit satisfaction. *Patient Educ. Couns.* **81**(Suppl), S94–S99 (2010). 20880656 10.1016/j.pec.2010.08.022 PMC2993845

[52] Li, J. et al. Communication needs of cancer patients and/or caregivers: A critical literature review. *J. Oncol.* **2020**, 7432849 (2020). 32454826 10.1155/2020/7432849 PMC7229568

[53] Wang, Y. et al. Communication between caregivers of adults with cancer and healthcare professionals: A review of communication experiences, associated factors, outcomes, and interventions. *Curr. Oncol. Rep.* **26**, 773–783 (2024). 38777979 10.1007/s11912-024-01550-5 PMC12424330

[54] Mitchell, K. R. et al. Operationalizing patient-centered cancer care: A systematic review and synthesis of the qualitative literature on cancer patients’ needs, values, and preferences. *Psychooncology.* **29**, 1723–1733 (2020). 32715542 10.1002/pon.5500 PMC7901502

[55] Al-Kaabi, F. The effect of hospital environment on patient engagement and perceived experience: A case study approach. *Int. J. Soc. Sci. Curr. Future Res. Trends.* **23**, 121–141 (2025).

[56] Özönder Ünal, I. & Ordu, C. Decoding caregiver burden in cancer: Role of emotional health, rumination, and coping mechanisms. *Healthcare (Basel).* **11**(19), 2700 (2023). 37830736 10.3390/healthcare11192700 PMC10573024

[57] Litzelman, K. Caregiver well-being and the quality of cancer care. *Semin. Oncol. Nurs.* **35**, 348–353 (2019). 31229346 10.1016/j.soncn.2019.06.006 PMC6728914

[58] Wang, T. et al. Unmet care needs of advanced cancer patients and their informal caregivers: A systematic review. *BMC Palliat Care.* **17**, 96 (2018). 30037346 10.1186/s12904-018-0346-9 PMC6057056

[59] Lis, C. G., Rodeghier, M., Grutsch, J. F. & Gupta, D. Distribution and determinants of patient satisfaction in oncology with a focus on health related quality of life. *BMC Health Serv. Res.* **9**, 190 (2009). 19845942 10.1186/1472-6963-9-190 PMC2770467

[60] Nembhard, I. M., David, G., Ezzeddine, I., Betts, D. & Radin, J. A systematic review of research on empathy in health care. *Health Serv. Res.* **58**, 250–263 (2023). 35765156 10.1111/1475-6773.14016 PMC10012244

[61] Wang, X., Wang, R., Sheng, F. & Chen, L. The effects of empathy by caregivers on healthcare service satisfaction. *Front Psychol.* **13**, 912076 (2022). 36275215 10.3389/fpsyg.2022.912076 PMC9582974

[62] Gomez-Cano, M., Lyratzopoulos, G. & Abel, G. A. Patient experience drivers of overall satisfaction with care in cancer patients: Evidence from responders to the English cancer patient experience survey. *J. Patient Exp.* **7**, 758–765 (2020). 33294612 10.1177/2374373519889435 PMC7705845

[63] Stiefel, F. et al. Communication and support of patients and caregivers in chronic cancer care: ESMO clinical practice guideline. *ESMO Open.* **9**, 103496 (2024). 39089769 10.1016/j.esmoop.2024.103496 PMC11360426

